# Cyclo­hexa­naminium 3,4,5,6-tetra­chloro-2-(meth­oxy­carbon­yl)benzoate

**DOI:** 10.1107/S1600536811008531

**Published:** 2011-03-12

**Authors:** Jian Li

**Affiliations:** a Department of Chemistry and Chemical Engineering, Weifang University, Weifang 261061, People’s Republic of China

## Abstract

In the title compound, C_6_H_14_N^+^·C_9_H_3_Cl_4_O_4_^−^, the cyclo­hexane ring of the cation adopts a chair conformation. In the anion, the mean planes of the meth­oxy­carbonyl and carboxyl­ate groups form dihedral angles of 67.3 (3) and 55.7 (3)°, respectively, with the benzene ring. In the crystal, inter­molecular N—H⋯O hydrogen bonds connect the components into chains along [100].

## Related literature

For related structures, see: Li (2011 ▶); Liang (2008 ▶). 
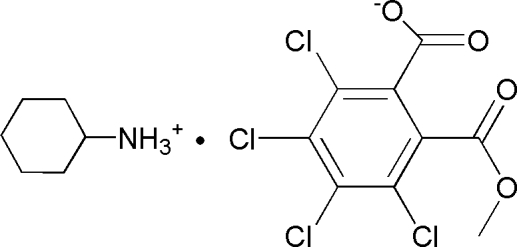


## Experimental

### 

#### Crystal data

C_6_H_14_N^+^·C_9_H_3_Cl_4_O_4_^−^
*M* *_r_* = 417.10
Triclinic, 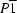

*a* = 5.9435 (4) Å
*b* = 11.3494 (12) Å
*c* = 14.3414 (15) Å
α = 90.755 (1)°
β = 97.901 (1)°
γ = 101.651 (2)°
*V* = 937.64 (15) Å^3^
*Z* = 2
Mo *K*α radiation
μ = 0.65 mm^−1^
*T* = 298 K
0.45 × 0.42 × 0.40 mm

#### Data collection

Bruker SMART CCD diffractometer
Absorption correction: multi-scan (*SADABS*; Bruker, 1997 ▶) *T*_min_ = 0.759, *T*_max_ = 0.781
4881 measured reflections
3239 independent reflections
2041 reflections with *I* > 2σ(*I*)
*R*_int_ = 0.017

#### Refinement

*R*[*F* ^2^ > 2σ(*F* ^2^)] = 0.050
*wR*(*F* ^2^) = 0.136
*S* = 1.03
3239 reflections
219 parameters
H-atom parameters constrained
Δρ_max_ = 0.22 e Å^−3^
Δρ_min_ = −0.32 e Å^−3^

### 

Data collection: *SMART* (Bruker, 1997 ▶); cell refinement: *SAINT* (Bruker, 1997 ▶); data reduction: *SAINT*; program(s) used to solve structure: *SHELXS97* (Sheldrick, 2008 ▶); program(s) used to refine structure: *SHELXL97* (Sheldrick, 2008 ▶); molecular graphics: *SHELXTL* (Sheldrick, 2008 ▶); software used to prepare material for publication: *SHELXTL*.

## Supplementary Material

Crystal structure: contains datablocks global, I. DOI: 10.1107/S1600536811008531/lh5210sup1.cif

Structure factors: contains datablocks I. DOI: 10.1107/S1600536811008531/lh5210Isup2.hkl

Additional supplementary materials:  crystallographic information; 3D view; checkCIF report

## Figures and Tables

**Table 1:** Hydrogen-bond geometry (Å, °)

*D*—H⋯*A*	*D*—H	H⋯*A*	*D*⋯*A*	*D*—H⋯*A*
N1—H1*A*⋯O4^i^	0.89	1.87	2.743 (4)	168
N1—H1*B*⋯O4^ii^	0.89	1.97	2.819 (3)	159
N1—H1*C*⋯O3^iii^	0.89	1.89	2.766 (4)	167

Symmetry codes: (i) 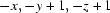; (ii) 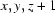; (iii) 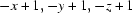.

**Fig. 1.**
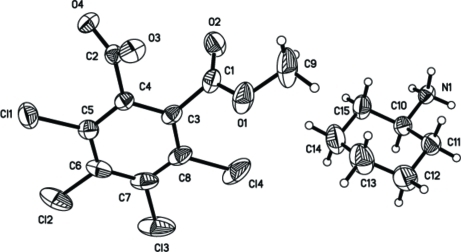
The asymmetric unit of (I), drawn with 30% probability ellipsoids.

**Fig. 2.**
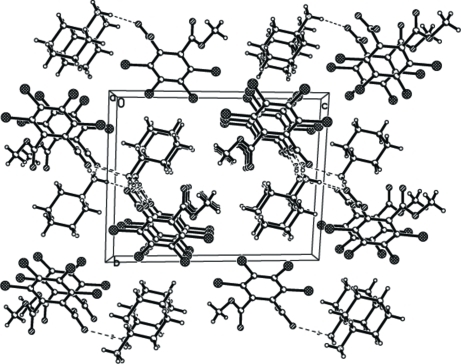
The crystal packing of (I), viewed along the a axis. Hydrogen bonds are indicated by dashed lines.

## supplementary crystallographic information

### Comment

From our reaction (see experimental section) we expected to form 4,5,6,7-tetrachloro-2-propylisoindoline-1,3-dione but instead formed the title compound. This may have happened because of the short time and cool temperature for the reaction. The asymmetric unit of the title compound (I) contains one cyclohexanaminium cation and one 3,4,5,6-tetrachloro-2-(methoxycarbonyl)benzoate anion (Fig. 1). The cyclohexane ring of the cation adopts a chair conformation. In the anion, the mean planes of the methoxycarbonyl and carboxylate groups form dihedral angles of 112.7 (3) and 55.7 (3) °, respectively with the benzene ring. The bond lengths and angles are in agreement with those which are related in in ethylammonium 2-(methoxycarbonyl)-3,4,5,6-tetrabromobenzoate methanol solvate (Li, 2011) and in ethane-1,2-diammonium bis(2-(methoxycarbonyl)-3,4,5,6-tetrabromobenzoate) methanol solvate (Liang, 2008). In the crystal, intermolecular N—H···O hydrogen bonds connect the components of the structure to form one-dimensional chains along [100] (Fig. 2).

### Experimental

A mixture of 4,5,6,7-tetrachloroisobenzofuran-1,3-dione (2.86 g, 0.01 mol) and methanol (15 ml) was refluxed for 0.5 h. Then cyclohexanamine (0.99 g, 0.01 mol) was added to the above solution and mixed for 20 min at room temperature. The solution was kept at room temperature for 5 d. Natural evaporation gave colourless single crystals of the title compound, suitable for X-ray analysis.

### Refinement

H atoms were initially located in difference maps and then refined in a riding model with C—H = 0.96–0.98 Å, N—H = 0.89 Å and *U*_iso_(H) = 1.2*U*_eq_(C) or 1.5*U*_eq_(N, methyl C).

### Crystal data

**Table:**

C_6_H_14_N^+^·C_9_H_3_Cl_4_O_4_^−^	*Z* = 2
*M**_r_* = 417.10	*F*(000) = 428
Triclinic, *P*1	*D*_x_ = 1.477 Mg m^−^^3^
Hall symbol: -P 1	Mo *K*α radiation, λ = 0.71073 Å
*a* = 5.9435 (4) Å	Cell parameters from 1650 reflections
*b* = 11.3494 (12) Å	θ = 2.3–24.6°
*c* = 14.3414 (15) Å	µ = 0.65 mm^−^^1^
α = 90.755 (1)°	*T* = 298 K
β = 97.901 (1)°	Block, colorless
γ = 101.651 (2)°	0.45 × 0.42 × 0.40 mm
*V* = 937.64 (15) Å^3^	

### Data collection

**Table:**

Bruker SMART CCD diffractometer	3239 independent reflections
Radiation source: fine-focus sealed tube	2041 reflections with *I* > 2σ(*I*)
graphite	*R*_int_ = 0.017
φ and ω scans	θ_max_ = 25.0°, θ_min_ = 2.3°
Absorption correction: multi-scan (*SADABS*; Bruker, 1997)	*h* = −7→6
*T*_min_ = 0.759, *T*_max_ = 0.781	*k* = −9→13
4881 measured reflections	*l* = −16→16

### Refinement

**Table:**

Refinement on *F*^2^	Primary atom site location: structure-invariant direct methods
Least-squares matrix: full	Secondary atom site location: difference Fourier map
*R*[*F*^2^ > 2σ(*F*^2^)] = 0.050	Hydrogen site location: inferred from neighbouring sites
*wR*(*F*^2^) = 0.136	H-atom parameters constrained
*S* = 1.03	*w* = 1/[σ^2^(*F*_o_^2^) + (0.0463*P*)^2^ + 0.7109*P*] where *P* = (*F*_o_^2^ + 2*F*_c_^2^)/3
3239 reflections	(Δ/σ)_max_ = 0.001
219 parameters	Δρ_max_ = 0.22 e Å^−^^3^
0 restraints	Δρ_min_ = −0.32 e Å^−^^3^

### Special details

**Table:**

Geometry. All e.s.d.'s (except the e.s.d. in the dihedral angle between two l.s. planes) are estimated using the full covariance matrix. The cell e.s.d.'s are taken into account individually in the estimation of e.s.d.'s in distances, angles and torsion angles; correlations between e.s.d.'s in cell parameters are only used when they are defined by crystal symmetry. An approximate (isotropic) treatment of cell e.s.d.'s is used for estimating e.s.d.'s involving l.s. planes.
Refinement. Refinement of *F*^2^ against ALL reflections. The weighted *R*-factor *wR* and goodness of fit *S* are based on *F*^2^, conventional *R*-factors *R* are based on *F*, with *F* set to zero for negative *F*^2^. The threshold expression of *F*^2^ > σ(*F*^2^) is used only for calculating *R*-factors(gt) *etc*. and is not relevant to the choice of reflections for refinement. *R*-factors based on *F*^2^ are statistically about twice as large as those based on *F*, and *R*- factors based on ALL data will be even larger.

### Fractional atomic coordinates and isotropic or equivalent isotropic displacement parameters (Å^2^)

**Table:**

	*x*	*y*	*z*	*U*_iso_*/*U*_eq_	
Cl1	0.0817 (2)	0.80648 (10)	0.03785 (7)	0.0826 (4)	
Cl2	−0.1463 (2)	0.97241 (11)	0.14937 (11)	0.1099 (5)	
Cl3	−0.0240 (3)	1.00178 (11)	0.36695 (12)	0.1385 (7)	
Cl4	0.3042 (3)	0.85179 (12)	0.47324 (8)	0.1157 (5)	
N1	0.2196 (4)	0.5077 (3)	0.90782 (18)	0.0543 (7)	
H1A	0.0689	0.4744	0.8983	0.081*	
H1B	0.2651	0.5258	0.9690	0.081*	
H1C	0.2996	0.4561	0.8883	0.081*	
O1	0.6531 (5)	0.7188 (3)	0.40242 (19)	0.0921 (10)	
O2	0.3683 (5)	0.5620 (3)	0.3543 (2)	0.0804 (8)	
O3	0.5961 (4)	0.6704 (2)	0.16858 (18)	0.0648 (7)	
O4	0.2495 (4)	0.5752 (2)	0.09970 (15)	0.0538 (6)	
C1	0.4495 (6)	0.6663 (4)	0.3552 (2)	0.0596 (9)	
C2	0.3833 (6)	0.6552 (3)	0.1529 (2)	0.0440 (7)	
C3	0.3263 (6)	0.7536 (3)	0.3036 (2)	0.0521 (8)	
C4	0.2755 (5)	0.7423 (3)	0.2053 (2)	0.0452 (8)	
C5	0.1354 (6)	0.8134 (3)	0.1590 (2)	0.0528 (8)	
C6	0.0384 (7)	0.8919 (3)	0.2082 (3)	0.0665 (11)	
C7	0.0916 (8)	0.9040 (3)	0.3046 (3)	0.0759 (12)	
C8	0.2351 (7)	0.8370 (3)	0.3518 (3)	0.0673 (11)	
C9	0.7685 (9)	0.6398 (6)	0.4622 (4)	0.125 (2)	
H9A	0.6549	0.5838	0.4896	0.188*	
H9B	0.8763	0.6871	0.5113	0.188*	
H9C	0.8503	0.5966	0.4251	0.188*	
C10	0.2615 (6)	0.6191 (3)	0.8543 (3)	0.0621 (10)	
H10	0.1572	0.6702	0.8708	0.075*	
C11	0.5086 (7)	0.6870 (4)	0.8821 (3)	0.0747 (11)	
H11A	0.6150	0.6355	0.8709	0.090*	
H11B	0.5344	0.7096	0.9488	0.090*	
C12	0.5552 (9)	0.7989 (4)	0.8256 (4)	0.1079 (17)	
H12A	0.4623	0.8545	0.8431	0.130*	
H12B	0.7174	0.8381	0.8409	0.130*	
C13	0.4999 (10)	0.7705 (5)	0.7218 (4)	0.121 (2)	
H13A	0.6065	0.7235	0.7028	0.145*	
H13B	0.5208	0.8449	0.6885	0.145*	
C14	0.2526 (10)	0.7006 (5)	0.6948 (4)	0.1153 (19)	
H14A	0.1452	0.7514	0.7063	0.138*	
H14B	0.2261	0.6781	0.6281	0.138*	
C15	0.2075 (8)	0.5892 (4)	0.7506 (3)	0.0829 (13)	
H15A	0.0459	0.5491	0.7351	0.100*	
H15B	0.3023	0.5343	0.7336	0.100*	

### Atomic displacement parameters (Å^2^)

**Table:**

	*U*^11^	*U*^22^	*U*^33^	*U*^12^	*U*^13^	*U*^23^
Cl1	0.0978 (8)	0.0969 (8)	0.0616 (6)	0.0438 (7)	0.0045 (5)	0.0168 (5)
Cl2	0.1097 (10)	0.0726 (7)	0.1708 (13)	0.0512 (7)	0.0501 (9)	0.0356 (8)
Cl3	0.2122 (17)	0.0708 (8)	0.1644 (14)	0.0421 (9)	0.1197 (13)	−0.0101 (8)
Cl4	0.1700 (14)	0.1050 (9)	0.0601 (7)	−0.0151 (9)	0.0422 (8)	−0.0307 (6)
N1	0.0421 (16)	0.0685 (19)	0.0515 (16)	0.0113 (14)	0.0050 (13)	−0.0072 (14)
O1	0.0748 (19)	0.109 (2)	0.0676 (18)	−0.0237 (17)	−0.0154 (15)	0.0083 (17)
O2	0.0749 (19)	0.0667 (18)	0.085 (2)	−0.0021 (15)	−0.0154 (15)	0.0157 (15)
O3	0.0399 (14)	0.0735 (17)	0.0796 (17)	0.0094 (12)	0.0088 (12)	−0.0134 (13)
O4	0.0450 (13)	0.0612 (14)	0.0530 (13)	0.0098 (11)	0.0028 (11)	−0.0159 (11)
C1	0.060 (2)	0.070 (3)	0.0400 (19)	−0.005 (2)	0.0039 (17)	0.0014 (18)
C2	0.047 (2)	0.0497 (19)	0.0373 (17)	0.0120 (16)	0.0086 (15)	0.0040 (15)
C3	0.057 (2)	0.0472 (19)	0.0468 (19)	−0.0069 (16)	0.0145 (16)	−0.0050 (15)
C4	0.0432 (18)	0.0445 (18)	0.0466 (18)	0.0042 (15)	0.0092 (14)	−0.0022 (14)
C5	0.055 (2)	0.050 (2)	0.055 (2)	0.0082 (17)	0.0171 (17)	0.0047 (16)
C6	0.073 (3)	0.041 (2)	0.094 (3)	0.0166 (18)	0.037 (2)	0.012 (2)
C7	0.101 (3)	0.045 (2)	0.090 (3)	0.010 (2)	0.054 (3)	−0.006 (2)
C8	0.090 (3)	0.053 (2)	0.056 (2)	−0.008 (2)	0.033 (2)	−0.0106 (18)
C9	0.085 (4)	0.173 (6)	0.091 (4)	−0.006 (4)	−0.035 (3)	0.038 (4)
C10	0.053 (2)	0.066 (2)	0.067 (2)	0.0155 (19)	0.0009 (18)	−0.0006 (19)
C11	0.061 (3)	0.071 (3)	0.083 (3)	0.004 (2)	−0.008 (2)	0.007 (2)
C12	0.093 (4)	0.084 (3)	0.129 (5)	−0.006 (3)	−0.013 (3)	0.026 (3)
C13	0.115 (5)	0.116 (4)	0.117 (5)	−0.006 (4)	0.002 (4)	0.053 (4)
C14	0.117 (4)	0.121 (4)	0.091 (4)	0.006 (4)	−0.021 (3)	0.038 (3)
C15	0.077 (3)	0.091 (3)	0.069 (3)	0.003 (2)	−0.011 (2)	0.010 (2)

### Geometric parameters (Å, °)

**Table:**

Cl1—C5	1.721 (3)	C9—H9A	0.9600
Cl2—C6	1.710 (4)	C9—H9B	0.9600
Cl3—C7	1.718 (4)	C9—H9C	0.9600
Cl4—C8	1.732 (4)	C10—C15	1.497 (5)
N1—C10	1.483 (4)	C10—C11	1.511 (5)
N1—H1A	0.8900	C10—H10	0.9800
N1—H1B	0.8900	C11—C12	1.514 (6)
N1—H1C	0.8900	C11—H11A	0.9700
O1—C1	1.323 (4)	C11—H11B	0.9700
O1—C9	1.454 (5)	C12—C13	1.496 (7)
O2—C1	1.185 (4)	C12—H12A	0.9700
O3—C2	1.230 (4)	C12—H12B	0.9700
O4—C2	1.250 (4)	C13—C14	1.518 (7)
C1—C3	1.492 (5)	C13—H13A	0.9700
C2—C4	1.523 (4)	C13—H13B	0.9700
C3—C4	1.399 (4)	C14—C15	1.503 (6)
C3—C8	1.400 (5)	C14—H14A	0.9700
C4—C5	1.386 (4)	C14—H14B	0.9700
C5—C6	1.388 (5)	C15—H15A	0.9700
C6—C7	1.374 (6)	C15—H15B	0.9700
C7—C8	1.371 (6)		
			
C10—N1—H1A	109.5	N1—C10—C15	110.3 (3)
C10—N1—H1B	109.5	N1—C10—C11	109.9 (3)
H1A—N1—H1B	109.5	C15—C10—C11	111.8 (3)
C10—N1—H1C	109.5	N1—C10—H10	108.2
H1A—N1—H1C	109.5	C15—C10—H10	108.2
H1B—N1—H1C	109.5	C11—C10—H10	108.2
C1—O1—C9	114.8 (3)	C10—C11—C12	110.3 (3)
O2—C1—O1	124.7 (4)	C10—C11—H11A	109.6
O2—C1—C3	122.5 (3)	C12—C11—H11A	109.6
O1—C1—C3	112.7 (3)	C10—C11—H11B	109.6
O3—C2—O4	126.4 (3)	C12—C11—H11B	109.6
O3—C2—C4	115.8 (3)	H11A—C11—H11B	108.1
O4—C2—C4	117.8 (3)	C13—C12—C11	112.0 (4)
C4—C3—C8	118.9 (3)	C13—C12—H12A	109.2
C4—C3—C1	119.3 (3)	C11—C12—H12A	109.2
C8—C3—C1	121.3 (3)	C13—C12—H12B	109.2
C5—C4—C3	118.9 (3)	C11—C12—H12B	109.2
C5—C4—C2	122.4 (3)	H12A—C12—H12B	107.9
C3—C4—C2	118.7 (3)	C12—C13—C14	111.5 (5)
C4—C5—C6	121.4 (3)	C12—C13—H13A	109.3
C4—C5—Cl1	119.8 (2)	C14—C13—H13A	109.3
C6—C5—Cl1	118.8 (3)	C12—C13—H13B	109.3
C7—C6—C5	119.4 (4)	C14—C13—H13B	109.3
C7—C6—Cl2	120.3 (3)	H13A—C13—H13B	108.0
C5—C6—Cl2	120.3 (3)	C15—C14—C13	111.0 (4)
C8—C7—C6	120.2 (3)	C15—C14—H14A	109.4
C8—C7—Cl3	119.6 (3)	C13—C14—H14A	109.4
C6—C7—Cl3	120.3 (4)	C15—C14—H14B	109.4
C7—C8—C3	121.2 (3)	C13—C14—H14B	109.4
C7—C8—Cl4	120.6 (3)	H14A—C14—H14B	108.0
C3—C8—Cl4	118.2 (3)	C10—C15—C14	111.3 (4)
O1—C9—H9A	109.5	C10—C15—H15A	109.4
O1—C9—H9B	109.5	C14—C15—H15A	109.4
H9A—C9—H9B	109.5	C10—C15—H15B	109.4
O1—C9—H9C	109.5	C14—C15—H15B	109.4
H9A—C9—H9C	109.5	H15A—C15—H15B	108.0
H9B—C9—H9C	109.5		
			
C9—O1—C1—O2	−5.2 (6)	Cl1—C5—C6—Cl2	4.3 (4)
C9—O1—C1—C3	173.5 (4)	C5—C6—C7—C8	−1.5 (6)
O2—C1—C3—C4	−63.1 (5)	Cl2—C6—C7—C8	178.3 (3)
O1—C1—C3—C4	118.2 (3)	C5—C6—C7—Cl3	179.4 (3)
O2—C1—C3—C8	108.9 (4)	Cl2—C6—C7—Cl3	−0.9 (5)
O1—C1—C3—C8	−69.8 (4)	C6—C7—C8—C3	−1.3 (6)
C8—C3—C4—C5	−0.5 (5)	Cl3—C7—C8—C3	177.9 (3)
C1—C3—C4—C5	171.7 (3)	C6—C7—C8—Cl4	179.8 (3)
C8—C3—C4—C2	177.4 (3)	Cl3—C7—C8—Cl4	−1.0 (5)
C1—C3—C4—C2	−10.4 (4)	C4—C3—C8—C7	2.3 (5)
O3—C2—C4—C5	122.9 (3)	C1—C3—C8—C7	−169.7 (3)
O4—C2—C4—C5	−58.1 (4)	C4—C3—C8—Cl4	−178.8 (2)
O3—C2—C4—C3	−54.9 (4)	C1—C3—C8—Cl4	9.1 (5)
O4—C2—C4—C3	124.1 (3)	N1—C10—C11—C12	178.3 (4)
C3—C4—C5—C6	−2.3 (5)	C15—C10—C11—C12	55.4 (5)
C2—C4—C5—C6	179.8 (3)	C10—C11—C12—C13	−54.7 (6)
C3—C4—C5—Cl1	176.9 (2)	C11—C12—C13—C14	54.7 (6)
C2—C4—C5—Cl1	−1.0 (4)	C12—C13—C14—C15	−54.6 (7)
C4—C5—C6—C7	3.3 (5)	N1—C10—C15—C14	−179.0 (4)
Cl1—C5—C6—C7	−175.9 (3)	C11—C10—C15—C14	−56.4 (5)
C4—C5—C6—Cl2	−176.5 (3)	C13—C14—C15—C10	55.3 (6)

### Hydrogen-bond geometry (Å, °)

**Table:**

*D*—H···*A*	*D*—H	H···*A*	*D*···*A*	*D*—H···*A*
N1—H1A···O4^i^	0.89	1.87	2.743 (4)	168
N1—H1B···O4^ii^	0.89	1.97	2.819 (3)	159
N1—H1C···O3^iii^	0.89	1.89	2.766 (4)	167

Symmetry codes: (i) −*x*, −*y*+1, −*z*+1; (ii) *x*, *y*, *z*+1; (iii) −*x*+1, −*y*+1, −*z*+1.
